# Social support status and associated factors among people living with HIV/AIDS in Kunming city, China

**DOI:** 10.1186/s12889-021-11253-2

**Published:** 2021-07-17

**Authors:** Yi Li, Xiao-Wen Zhang, Bin Liao, Jun Liang, Wen-Jie He, Jun Liu, Yao Yang, Yun-Hai Zhang, Tao Ma, Jing-Ying Wang

**Affiliations:** 1 Department of AIDS and Sexually Transmitted Disease Control and Prevention, Kunming Center for Disease Control and Prevention, 1.4# Zi Yun Road, Dian Chi Lv You Du Jia District, Kunming, Yunnan Province China; 2 Kunming Municipal Health Commission, 2.4 / F, building 8, municipal administration center, 1# Jinxiu street, Chenggong New District, Kunming, Yunnan Province China

**Keywords:** HIV/AIDS, Social support, Associated factors

## Abstract

**Background:**

People living with HIV/AIDS not only require effective treatment for the alleviation of physical discomfort but also require social support to help them address difficulties in life and relieve their psychological anxiety and uneasiness. The social support network is of tremendous importance in helping people living with HIV/AIDS maintain good physical and mental health. This study aims to analyse the social support status among people living with HIV/AIDS in Kunming and explore associated factors.

**Method:**

The Social Support Rating Scale (SSRS) was used, and a questionnaire survey was conducted using convenience sampling to select people living with HIV/AIDS from 14 counties of Kunming. It collected information on general demographic information and social support status. Univariate and multivariate linear regression models were used to explore the associated factors.

**Results:**

A total of 990 valid questionnaires were completed. Data from all participants were analysed. Univariate analysis suggested that the factors associated with social support may include marital status, monthly income, and antiretroviral therapy. On the other hand, factors including monthly income and antiretroviral therapy accounted for the social support total score in the multivariate analysis.

**Conclusion:**

Social support among people living with HIV/AIDS in Kunming was generally low. This study identified a number of factors associated with social support among people living with HIV/AIDS. Based on our findings, appropriate interventions should be introduced to provide social support for those living with HIV/AIDS.

## Background

Human immunodeficiency virus (HIV) damages patients’ normal physiological functioning, thus putting them in a difficult life and economic situation. Social discrimination causes people living with HIV/AIDS to suffer from difficulties in interpersonal relationships and social environments. For patients whose early symptoms were not obvious, their psychological impact was far greater than their physical pain [1]. Therefore, people living with HIV/AIDS not only require effective treatment to alleviate their physical discomfort but also require social support to help them solve their life difficulties and relieve their psychological anxiety and uneasiness.

Social support refers to the system of spiritual or material support from all aspects of society, including parents, relatives and friends [2]. As a multifunctional social system, social support improves an individual’s physical and mental health. It plays an important role in coping with and recovering from diseases [3]. Social support could moderate the negative effects of stressful events [4], and it is one of the most effective ways to cope with stress [5]. Research indicates that the social support network is of great significance to help people living with HIV/AIDS maintain good physical and mental health [6]. Therefore, it is important to research the current social support system among people living with HIV/AIDS and improve their social support status. Our study aims to determine the factors influencing social support for people living with HIV/AIDS in Kunming and provide a basis for developing targeted interventions in the future.

## Methods

### Study design and objective

HIV/AIDS cases were selected from the National Comprehensive HIV/AIDS Information System, with follow-up conducted until August 31, 2018. Inclusion criteria were as follows: (1) age 18 to 75 years; (2) no serious diseases or mental disorders; (3) consent to cooperate with the investigation; and (4) all questions answered truthfully and completely.

A convenience sampling method was used to select participants. The participants could be contacted by Centers for Disease Control and Prevention (CDC) staff from 14 counties in Kunming. Before the interviews, the Kunming Centers for Disease Control and Prevention (KMCDC) staff conducted unified training for CDC investigators in the 14 counties. The informed consent obtained from participants was verbal. Then, face-to-face interviews were conducted using the general demographic characteristics questionnaire and the Social Support Rating Scale (SSRS). The general questionnaire mainly include demographic characteristics (sex, age, nationality, marital status, etc) and monthly income, HIV transmission route, therapy status during investigation, etc.

The Social Support Rating Scale (SSRS) has been used in many domestic studies. The Cronbach’s α coefficient is 0.896, which indicates good reliability and validity [7]. The SSRS which used in the questionnaire has previously been firstly developed by Xiao Shui Yuan in 1986 [7]. It has been used to survey social support status. There are 10 items across three dimensions: objective support, subjective support and the utilization of support. Objective support refers to objective, realistic and visible support, including material and behavioral help [7]. Subjective social support refers to an individual’s subjective feelings and evaluation of the support he or she receives from members of his or her social support network [8]. The utilization of social support includes: the way to talk and seek help when encountering trouble, and the frequency of participating in group activities [9]. The total score was the summary of scores for each item. The higher the score was, the better the social support status. The scale ware application among people living with HIV/AIDS in some studies in China, It shows that the scale has certain applicability [10].

### Statistical analysis

Data were input using EpiData 3.1 software and double-entered into the database. SPSS 23.0 software was used for statistical analysis. Univariate factor analysis was performed by t-test or ANOVA, and *P* < 0.05 was considered statistically significant. Then, the meaningful factors in univariate factor analysis were put into multivariate linear regression analysis. The level of significance was defined as *P* < 0.05.

## Results

### Survey response

A total of 932 participants were administered the survey, and 900 valid questionnaires were returned. Thus, the effective response rate was 96.6%.

### Social demographic characteristics and the general situation

There were 716 males (72.3%) and 274 females (27.7%), with an average age of 40.46 ± 12.83 years. Those with Han nationality comprised the majority of the participants, accounting for 81.9% of the total sample. A total of 639 participants (64.5%) with an education level of junior high school and below. Most of them (79.4%) had a monthly income of less than 3000 CNY. With respect to marital status, most participants were unmarried or married/cohabiting (74.7%), the proportion of participants were divorced account for 31.0%.

The total score for social support among people living with HIV/AIDS was 26.45 ± 8.70, the objective support score was 5.33 ± 3.79, the subjective support score was 15.62 ± 4.91, and the degree of utilization of social support score was 5.50 ± 2.10.

### Univariate analysis of the demographic characteristics

Univariate analysis was carried out on the social demographic factors of people living with HIV/AIDS, and comparisons among groups were performed using t-tests or ANOVA (*P* < 0.05). Marital status, monthly income, infection route and univariate therapy are the factors influencing objective support. Those who are married or cohabiting, whose monthly income is 3000 yuan or above, whose infection route is male-to-male transmission and who are undergoing univariate therapy have higher scores for objective support. Sex and marital status are the factors influencing subjective support among people living with HIV/AIDS. Participants who are female, married or cohabiting have higher scores for subjective support. Sex, marital status, age, ethnicity, occupation, and infection route are the factors influencing the utilization of support. The age group is 29 years old or below, and the infection route is male-to-male transmission, resulting in a higher score for utilization of support. Marital status, monthly income and the receipt of univariate therapy are the factors influencing the total score for social support. HIV/AIDS patients who are married or cohabit, who have higher income and who receive univariate therapy have higher total social scores (Table 1).

**Table 1:** Comparison of social support scores among HIV/AIDS patients with different Socio-demographic characteristics (X ± S)

Item	n	Scores			
		Objective support	Subjective support	Degree of utilization of social support	Total score of social support
Sex					
Male	716	5.21 ± 3.89	15.43 ± 4.86	5.53 ± 2.14	26.17 ± 8.86
Female	274	5.64 ± 3.51	16.13 ± 5.02	5.42 ± 2.01	27.19 ± 8.26
(t,p)		−1.652,0.099	−2.014,0.044	0.728,0.467	−1.700,0.090
Age					
≤ 20	18	6.44 ± 3.11	16.00 ± 4.38	6.33 ± 2.35	28.78 ± 6.37
20–29	188	5.31 ± 4.00	14.95 ± 4.40	5.76 ± 1.98	26.02 ± 8.10
30–39	293	5.31 ± 3.87	15.80 ± 5.21	5.51 ± 2.18	26.61 ± 9.16
40–49	291	4.98 ± 3.57	15.81 ± 4.91	5.53 ± 2.16	26.32 ± 8.64
50–59	114	5.66 ± 3.79	15.84 ± 5.17	5.08 ± 1.92	26.58 ± 9.03
≥ 60	86	5.99 ± 3.82	15.45 ± 4.66	5.21 ± 2.02	26.65 ± 8.69
(F,p)		1.516,0.182	0.948,0.449	2.416,0.034	0.396,0.852
Ethnicity					
Han nationality	811	5.31 ± 3.77	15.63 ± 4.82	5.41 ± 1.96	26.35 ± 8.47
Ethnic minority	179	5.42 ± 3.90	15.56 ± 5.32	5.92 ± 2.62	26.91 ± 9.69
(t,p)		−0.342,0.732	0.168,0.866	−2.469,0.014	−0.704,0.482
Educational level					
Primary school or below	259	5.58 ± 3.60	15.51 ± 4.67	5.51 ± 2.38	26.59 ± 8.79
Junior high school	380	4.96 ± 3.56	15.52 ± 5.05	5.33 ± 2.03	25.81 ± 8.65
High school	149	5.35 ± 3.92	16.23 ± 4.98	5.58 ± 2.17	27.16 ± 8.52
College or above	202	5.71 ± 4.28	15.50 ± 4.90	5.75 ± 1.79	26.97 ± 8.82
(F/welch,p)		2.321,0.075	0.896,0.443	2.190,0.089	1.282,0.279
Marital status					
Single	298	6.38 ± 3.49	17.46 ± 5.24	5.48 ± 2.03	29.32 ± 8.45
Married/cohabiting	374	4.99 ± 3.79	14.90 ± 4.23	5.70 ± 2.22	25.60 ± 8.07
Divorced	279	4.78 ± 3.92	14.72 ± 4.87	5.30 ± 2.00	24.80 ± 9.02
Bereaved wife or husband	39	4.51 ± 3.52	14.92 ± 5.13	5.15 ± 2.17	24.59 ± 9.09
(F/welch,p)		11.452,0.000	18.787,0.000	2.363,0.070	16.675,0.000
Occupation					
Yes	293	5.66 ± 4.29	15.62 ± 4.94	5.73 ± 2.19	27.00 ± 8.92
No	697	5.20 ± 3.55	15.61 ± 4.86	5.41 ± 2.06	26.22 ± 8.60
(t,p)		1.616,0.107	0.024,0.981	2.219,0.027	1.281,0.200
Monthly income					
≤ 500	247	4.58 ± 3.16	15.55 ± 5.35	5.23 ± 2.67	25.96 ± 9.17
500–1000	116	5.09 ± 3.61	15.11 ± 4.77	5.10 ± 1.73	25.31 ± 7.91
1000–2000	236	5.64 ± 4.10	15.75 ± 5.18	5.11 ± 1.87	26.50 ± 9.28
2000–3000	206	5.47 ± 4.04	15.33 ± 4.10	5.20 ± 1.74	26.20 ± 7.97
≥ 3000	185	5.95 ± 3.82	16.18 ± 4.86	5.32 ± 1.97	28.05 ± 8.42
(F/welch,p)		5.008,0.001	1.226,0.299	1.995,0.105	2.489,0.043
Minimum living allowances					
Yes	188	4.81 ± 3.53	15.48 ± 5.11	5.41 ± 2.08	25.51 ± 8.31
No	802	4.45 ± 3.88	15.65 ± 4.87	5.57 ± 2.10	26.68 ± 8.78
(t,p)		−1.279,0.059	−0.439,0.661	−1.799,0.056	−1.661,0.097
HIV transmission route					
Male-to-male transmission	183	5.78 ± 4.25	15.17 ± 4.57	5.83 ± 1.80	26.78 ± 8.47
Heterosexual transmission	591	5.51 ± 3.80	15.86 ± 5.13	5.36 ± 2.04	26.73 ± 8.92
Injection drug abuse	196	4.32 ± 3.19	15.27 ± 4.52	5.63 ± 2.46	25.21 ± 8.28
Unknown	20	5.85 ± 2.66	15.90 ± 4.78	5.60 ± 2.35	27.35 ± 7.78
(F/welch,p)		7.693,0.000	1.344,0.259	3.167,0.028	1.688,0.168
Therapy status during investigation					
Never been treated	551	5.05 ± 3.52	15.41 ± 4.79	5.40 ± 2.24	25.86 ± 8.56
Dropped out	252	5.31 ± 3.64	15.82 ± 4.95	5.44 ± 2.04	26.57 ± 8.06
Group retained in care	187	6.21 ± 4.56	15.96 ± 5.19	5.89 ± 1.70	28.05 ± 9.72
(F/welch,p)		5.079,0.007	0.851,0.427	5.295,0.005	3.853,0.022

### Multivariate linear regression analysis

Taking the total scores for social support and the scores for each dimension as the dependent variable (Y), the factor with *P* < 0.05 was selected as the independent variable on the basis of univariate factor analysis. The multivariate linear stepwise regression analysis was conducted under the standard of 0.05 for entry level and 0.10 for rejection level. The results of multivariate stepwise regression analysis are shown in Table 2. The results show that females are favourable factors for subjective support. Marriage/cohabitation is a favourable factor for objective support. High monthly income and antiretroviral therapy are favourable factors for objective support and total scores for social support. Compared with injection drug use, male-to-male transmission of HIV/AIDS is associated with more objective support and utilization of support. Age and antiretroviral therapy are favourable factors in the utilization of support (Table 2).

**Table 2:** Multivariate linear regression analysis of social support among HIV/AIDS patients

All dimensions of social support	Associated factors	β	S.E	t	*P*
Objective support	Marital status	−0.061	0.135	4.505	0.000
	Monthly income	0.247	0.088	2.815	0.005
	HIV transmission route	0.291	0.101	2.878	0.004
	antiretroviral therapy	0.508	0.152	3.335	0.001
Subjective support	Sex	0.761	0.358	2.127	0.034
	Marital status	−0.129	0.177	−0.729	0.466
Degree of utilization of social support	Age	−0.115	0.057	−2.019	0.044
	Ethnicity	0.279	0.178	1.563	0.118
	Occupation	−0.211	0.156	−1.351	0.177
	HIV transmission route	−0.117	0.056	−2.087	0.037
	antiretroviral therapy	0.179	0.086	2.079	0.038
Total score of social support	Marital status	−0.245	0.311	−0.786	0.432
	Monthly income	0.390	0.196	1.992	0.047
	antiretroviral therapy	1.013	0.353	2.872	0.004

## Discussion

Our study found that the total score for social support for people living with HIV/AIDS was 12–57 (26.45 ± 8.70), which was lower than the domestic norm (34.56 ± 3.73). The difference was statistically significant as well (t = 29.308, *P* = 0.000), which was consistent with the results of Guo Zihan in Guangzhou [11]. The social support level among people living with HIV/AIDS was relatively low.

In this study, subjective support scores among females were higher than those among males. This may be because females are more inclined to share their unpleasant experiences with their relatives, friends or colleagues to reduce psychological pressure. Men are more likely to choose to endure setbacks or face difficulties, hoping to overcome and reduce their psychological burden through their own efforts. This reflects that we should pay more attention to males living with HIV/AIDS and encourage them to participate in various forms of activities and share their inner thoughts and feelings with others.

The lowest utilization of social support was among HIV/AIDS patients aged 50–59 years. Social support among older adults with HIV/AIDS mainly comes from their families. They may not have children around them, and their use of social support is relatively lower. The results of this study are consistent with those of Ma Li et al. [12]. We should encourage middle-aged and older HIV/AIDS patients to participate in healthy cultural activities and create a supportive social environment. To promote the physical and mental health of the middle-aged and older HIV/AIDS, full consideration should be given to the importance of social support, family support and psychological support for the elderly should be enhanced, their enthusiasm should be fully mobilized, they should participate in more social activities and their attitude towards life should be improved. Encourage them to seek help and support from their families and communities. In this way, infected people may be optimistic in facing HIV, actively cooperate with treatment, improve their quality of life, and prolong their life span.

The objective support score for married or cohabitant individuals was higher than that for unmarried, divorced or widowed HIV/AIDS patients, which was consistent with previous studies [13]. Spouses and family members are the main sources of social support for people living with HIV/AIDS. The help of spouses or family members of those with HIV/AIDS will help them develop more confidence in life. Social support from spouses and families of those with HIV/AIDS should be strengthened. For unmarried people, support mainly from family members, such as parents, brothers and sisters, should be an area of greater focus.

In this study, monthly income affected the scores on objective support and the total scores for social support. After patients develop AIDS, they may experience a decline in physical functioning, which is more likely to result in opportunistic infections and other AIDS-related infections. Then, they may experience increased medical costs. Due to the decline in the HIV/AIDS labour force and the lack of employment opportunities, all these situations affect their psychological status. Those with a higher income can shoulder the cost of therapy and have better physiological and psychological status. On the other hand, people with higher income tend to receive more support and help from their families.

Compared with injection drug abuse and heterosexual transmission, people who become infected with HIV/AIDS by male-to-male transmission have more objective support and utilization of support. There are few related reports in China. This may be attributed to the fact that they can supported by their homosexual partners. Because of the particular HIV infection route, drug addicts and sexually infected people seldom receive social support from family and friends. Social discrimination contributes to their isolation from society, which leads to decreased social support scores. We should establish a comprehensive care system and pay attention to the special groups of individuals with HIV/AIDS.

With comprehensive measures for AIDS prevention and control, people’s awareness of AIDS has constantly improved, and various anti-discrimination actions have been advocated to reduce the degree of social discrimination [14]. In addition, with the implementation of the “Four Frees and One Care” policy, the proportion of AIDS patients receiving free univariate therapy in China is relatively high, resulting in relatively high social support [15]. In this study, the HIV/AIDS patients who received univariate therapy received higher scores on objective support, the utilization of support and total social support than did those who did not receive univariate therapy or who dropped out of treatment, which reflected that national care and assistance policies such as univariate therapy played an important role in improving social support for HIV/AIDS. The research shows that nurses serve as an important social support system for individuals with HIV/AIDS and can provide the most direct and effective emotional and informational support [16]. Emotional support refers to an individual being respected and accepted by others. Informational support refers to helping people explain, understand and respond to problematic events [17]. Therefore, we should fully distribute the social support from medical and nursing staff to individuals with HIV/AIDS and create a positive social environment for AIDS prevention and control.

### Limitations

Our study has limitations. 1.This was a cross-sectional study with a small sample size. The causal relationship between factors and social support has not been confirmed, which may cause bias. Therefore, further studies are needed to demonstrate how these factors affect social support for people living with HIV/AIDS.2.Convenience sampling was used in this study may cause some selection bias. And multi-factor analysis was used to eliminate the influence of confounding factors including sex.

## Conclusion

This study identified that the social support level among people living with HIV/AIDS in Kunming was relatively lower than the domestic norm and that some factors affect social support among those living with HIV/AIDS. Based on our findings, appropriate measures should be introduced to increase social support. We should pay attention to those living with HIV/AIDS who are elderly, are injection drug users, are divorced and have a low economic level. The government should provide more financial support and strengthens AIDS-related medical services to improve social support for those living with HIV/AIDS.

## Data Availability

The data used in this study are available from the corresponding author upon reasonable request.

## Abbreviations

HIV: Human immunodeficiency virus
AIDS: Acquired immunodeficiency syndrome
SSRS: Social Support Rating Scale
ART: Antiretroviral therapy
